# Improved Forward Osmosis Performance of Thin Film Composite Membranes with Graphene Quantum Dots Derived from Eucalyptus Tree Leaves

**DOI:** 10.3390/nano12193519

**Published:** 2022-10-08

**Authors:** Haleema Saleem, Asif Saud, Nazmin Munira, Pei Sean Goh, Ahmad Fauzi Ismail, Hammadur Rahman Siddiqui, Syed Javaid Zaidi

**Affiliations:** 1 UNESCO Chair on Desalination and Water Treatment, Center for Advanced Materials, Qatar University, Doha P.O. Box 2713, Qatar; 2 Advanced Membrane Technology Research Centre, School of Chemical and Energy Engineering, Universiti Teknologi Malaysia, Johor Bahru 81310, Malaysia

**Keywords:** graphene quantum dots, eucalyptus trees, nanofiber membranes, forward osmosis, solution blow spinning

## Abstract

The major challenges in forward osmosis (FO) are low water flux, high specific reverse solute flux (SRSF), and membrane fouling. The present work addresses these problems by the incorporation of graphene quantum dots (GQDs) in the polyamide (PA) layer of thin-film composite (TFC) membranes, as well as by using an innovative polyethersulfone nanofiber support for the TFC membrane. The GQDs were prepared from eucalyptus leaves using a facile hydrothermal method that requires only deionized water, without the need for any organic solvents or reducing agents. The nanofiber support of the TFC membranes was prepared using solution blow spinning (SBS). The polyamide layer with GQDs was deposited on top of the nanofiber support through interfacial polymerization. This is the first study that reports the fouling resistance of the SBS-nanofiber-supported TFC membranes. The effect of various GQD loadings on the TFC FO membrane performance, its long-term FO testing, cleaning efficiency, and organic fouling resistance were analyzed. It was noted that the FO separation performance of the TFC membranes was improved with the incorporation of 0.05 wt.% GQDs. This study confirmed that the newly developed thin-film nanocomposite membranes demonstrated increased water flux and salt rejection, reduced SRSF, and good antifouling performance in the FO process.

## 1. Introduction

**Figure 1:**
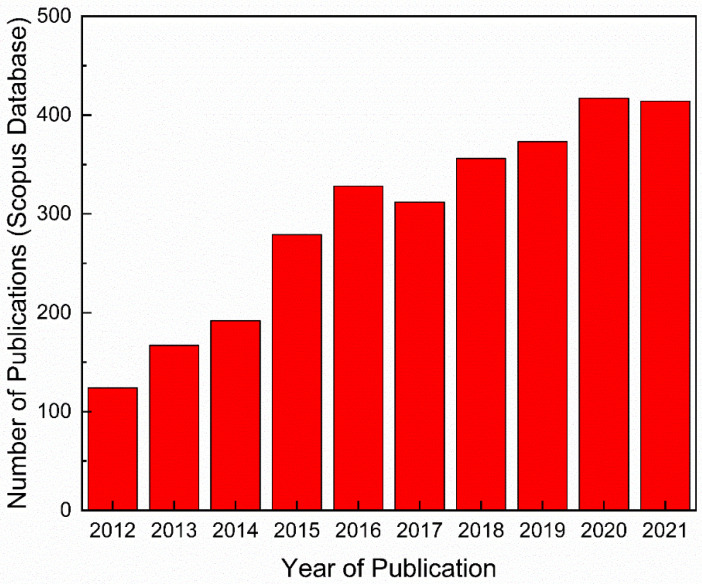
Number of publications related to “Forward osmosis”. Data obtained from the Scopus database (accessed on 16 July 2022).

The worldwide depletion of freshwater sources, climatic change, and the increased demand for fresh water due to population increases and rapid economic development are the major challenges that humankind currently faces. Desalination and wastewater treatment are the two most important potential solutions to the freshwater shortage issues [1,2]. The membrane-based separation technique is an ecofriendly and effective approach for freshwater production and for wastewater treatment [3,4]. Out of the different membrane-based water separation techniques, forward osmosis (FO) has turned out to be an efficient and innovative technology that has received significant attention from both the industrial and scientific sectors in the last decade [5,6]. From Figure 1, it can be noted that the number of studies related to “Forward osmosis” has increased over the last 10 years. These details were obtained from Scopus database, by identifying articles where the keyword is noted in the title, abstract, or keywords. Because of its interesting features, which include a lower hydraulic pressure requirement, reduced energy consumption, increased water recovery, simplicity, and satisfactory resistance against a wide range of pollutants [7], FO has the potential to be employed in several applications, such as wastewater treatment [8] and seawater and brackish water desalination [9,10], as well as power generation [11]. However, the lack of an ideal FO membrane that features a high water flux, a low solute flux, a minimum reverse solute flux (RSF), high rejection, high mechanical and chemical stability, and low fouling tendencies has hampered the commercial application of membrane-based FO technology [12,13]. Membrane fouling is the main disadvantage of FO, as it negatively impacts water productivity, the membrane lifespan, and operating expenses [14]. It has been proven that a more hydrophilic and thinner polyamide layer can enhance the water permeability as well as the anti-fouling properties of the thin-film composite (TFC) membrane [15,16]. Moreover, the reverse solute flux (RSF), i.e., the salt diffusion from the draw solution (DS) to the feed solution (FS), also has significant effects on water flux as well as membrane fouling. Membrane fouling involves organic and inorganic fouling, biofouling, and colloidal fouling [17]. For the organic fouling of salt-rejecting membranes, the decrease in the water flux is mainly due to the enhancement of the total hydraulic resistance caused by the organic fouling layer [18]. Additionally, organic fouling is strongly dependent upon particular physiological factors of the membrane, including membrane roughness, membrane materials, zeta potential, and hydrophilicity [18].

Nanomaterials are added to the polyamide (PA) active layer to form a thin-film nanocomposite (TFN) membrane with enhanced separation efficiency [19,20,21,22]. The concept of TFN was initially introduced by Jeong et al. in 2007 [23]. Many studies have confirmed that PA-TFN membranes can considerably improve the membrane’s properties, such as selectivity, permeability, fouling resistance, and stability, in several membrane-based separation processes [19,24,25,26,27]. The inclusion of hydrophilic nanomaterials into the support layer of a TFC FO membrane can improve the hydrophilicity of the membrane and reduce the tortuosity of the substrate. Considerable research has been carried out in the area of carbon-based nanomaterials, as evidenced by a huge number of research articles, published in various fields, which are related to membrane technology [28]. Graphene-based nanomaterials have received considerable attention for many cutting-edge applications [29,30,31] including water treatment, sensors, etc. [32,33,34,35,36]. Their extraordinary features make them one of the carbon-based materials most suitable for the development of membranes. Studies have confirmed that TFC FO membranes with graphene oxide showed higher water flux and improved hydrophilicity, as well as superior anti-fouling properties [37]. Out of the graphene-based materials, graphene quantum dots (GQDs) have exceptional structure-related properties, and they are both physically and chemically stable due to their intrinsic inert carbon properties [38]. Similar to carbon dots, which are suitable for use in membrane-based technologies [39], GQDs are also able to provide good properties to the membranes used in water treatment applications. In general, GQDs consist of limited graphene layers with a small size distribution, in the 2 to 20 nm range [40]. GQDs carry hydrophilic hydroxyl as well as carboxyl groups on the nanosheet’s edge, along with a hydrophobic aromatic ring on the nanosheet’s plane [41]. The GQDs possess high specific surface area, good biocompatibility, and plentiful edge sites for functionalization, and offer highly hydrophilic groups, as compared to graphene oxide. GQDs are used in different membrane-based separation processes, such as reverse osmosis [42], pervaporation [43], and forward osmosis [41], due to their superior properties. They can offer good hydrophilic properties to membranes, thereby improving their performance. When GQDs are added into the PA layer of TFC membranes, they have a uniform distribution, such that the GQD stretch in the selective layer is reduced. This results in the formation of a very smooth surface, which might be advantageous for the fouling resistance properties of the membranes. Moreover, the small size of the GQDs particles might lead to a thinner PA separation layer. Furthermore, the surface chemistry and the small size of GQDs assist in their appropriate dispersion within polymeric matrices, as well as in polar solvents such as water, which are vital properties for the application of GQDs in membrane fabrication and membrane-based separation processes [41]. All of the above-mentioned features provide the GQD-incorporated FO membranes with good performance abilities. A work by Xu, S. et al. [41] confirmed that the separation performance of TFC membranes in the FO process was improved with the incorporation of chemically prepared GQDs into the PA layer. Because of their interesting properties, it is desirable to prepare GQDs through different techniques, including microwave-assisted hydrothermal techniques [44], electrochemical techniques [45] such as cyclic voltammetry [46], ruthenium-catalyzed C60 transformation [47], and electron beam lithography [48]. GQDs are prepared from different sources, including carbon fibers [49], glucose [50], various forms of graphite [51], and multi-walled carbon nanotubes (MWCNTs) [52]. Preferably, the GQD preparation protocol should have minimum negative impacts on the environment and on human health. As such, the use of strong chemicals such as acids should be avoided [53]. Furthermore, the utilization of expensive instruments and costly materials, such as MWCNTs and ruthenium, might restrict its practical uses. Moreover, the reported quantum yield is normally low when using preparation techniques such as the electrochemical technique to synthesize GQDs. These limitations have prompted the preparation of GQDs from more renewable, sustainable, natural, and inexpensive sources, such as green plants, which are the foundation of the majority of the planet’s ecologies. Naturally occurring carbon-containing materials are receiving increased attention around the globe because of their exceptional chemical properties, physical properties, morphologies, and outstanding applications [54,55,56]. Even though different carbon species including candle soot, carbon black, coal, and carbon fibers [57,58] have been employed as a carbon feedstock for preparing GQDs in a sophisticated way, the above-mentioned carbon precursors are entirely associated with the non-renewable sources and might not be adequately accessible in the upcoming years [59,60]. Hence, it is essential to discover more sustainable carbon sources from renewable, sustainable, and natural resources. Recently, CNTs have been prepared from natural sources including palm [61], eucalyptus [62], and turpentine [63]. Eucalyptus tree extracts are mainly composed of hydrocarbons with lower oxygen content [64], and this appears to be a perfect precursor species for synthesizing GQDs. 

To overcome the present challenges in FO membranes and to additionally enhance the performance of FO membranes, i.e., to increase water flux and reduce the RSF, nanofibrous support with high porosity and a hydrophilicity nanofibrous substrate is recommended. Nanofibers have a higher potential in membrane-based separation technology due to their high porosity, submicron pore sizes, and large surface area to volume ratio, which promotes low production costs and simply tailored membrane thickness [65]. Various polymers, including polyether sulfone (PES), polysulfone (PSF), polyacrylonitrile (PAN), polyvinylidene fluoride (PVDF), and cellulose acetate (CA), are used to prepare nanofiber membranes (NFMs) [65]. Of these different polymers, PES has received more research consideration because of its multiple potential applications; this is due to its exceptional mechanical properties and superior environmental resistance and thermo-oxidative as well as thermal stability properties [66]. Presently, conventional technologies such as electrospinning (ES), melt blowing, and melt spinning are used for fiber production. Although the ES method is suitable for the commercial manufacturing of nanofibers, its low fiber production capacity is considered to be its main drawback [67]. Furthermore, the solvents compatible with the ES process are restricted by their dielectric constant; moreover, an extremely high voltage supply (up to 60 kV) is necessary for the ES process. The solution blow spinning (SBS) technique, which combines elements of both ES technologies and melt blowing, has been developed as a substitute for fiber production. SBS overcomes the low production rate of ES technology and the restricted choice of materials for melt blowing. This system is extremely simple, and the diameter of the nanofibers obtained is same as the diameter of nanofibers fabricated using ES [68]. 

In the current study, GQDs prepared from eucalyptus tree leaves were used as nanofillers for the PA layer of TFC membranes. The GQDs were prepared from leaf extracts following the procedure reported by Roy et al. [69]. This was the first attempt to synthesize GQDs from eucalyptus tree leaves using an eco-friendly method, without the use of any chemicals in its preparation. Additionally, the PES nanofiber support layer was prepared using the solution blow spinning method. Heat treatment of the NFM was performed to improve the mechanical properties and structure of the membrane. The interfacial polymerization (IP) process was performed over the support layers to produce PA layers with and without GQDs. The composition, morphology, and structure of the nanomaterial and the composition and water contact angle (CA) of the developed membranes were examined. Subsequently, the FO performance of the developed membranes was evaluated with respect to water flux, solute flux, specific reverse solute flux (SRSF), and salt rejection. The FO tests were performed using 0.1 M sodium chloride (NaCl) as the feed solution, and 1.5 M NaCl as the draw solution. These tests were performed using a crossflow bench scale FO system in the active layer facing feed solution (AL-FS) mode and the active layer facing draw solution (AL-DS) mode. The organic fouling resistance of the TFN membranes was also analyzed using a humic acid solution. This study is the first to investigate the fouling resistance of TFC membranes with solution-blown spun nanofiber support for FO applications.

## 2. Materials and Methods

### 2.1. Materials

The polyethersulfone (PES), N-methyl-2pyrrolidone, and toluene used for the nanofiber production were obtained from Sigma Aldrich, St. Louis, MO, USA. The trimesoyl chloride (TMC) (>98%), 1,3-phenylenediamine (MPD) (>99%), deionized water (DI), and n-hexane used for the interfacial polymerization process were purchased from Merck, Kenilworth, NJ, USA. The eucalyptus tree leaves were collected from the Qatar University campus. Furthermore, the sodium chloride (AR ACS 99.5%) used in FO testing and the humic acid (HA) used for the organic fouling resistance study were obtained from Nice Chemicals India, Kerala, India, and Sigma Aldrich, respectively. 

### 2.2. Preparation of GQDs from the Dry Leaves of Eucalyptus Trees Using DI Water

**Figure 2:**
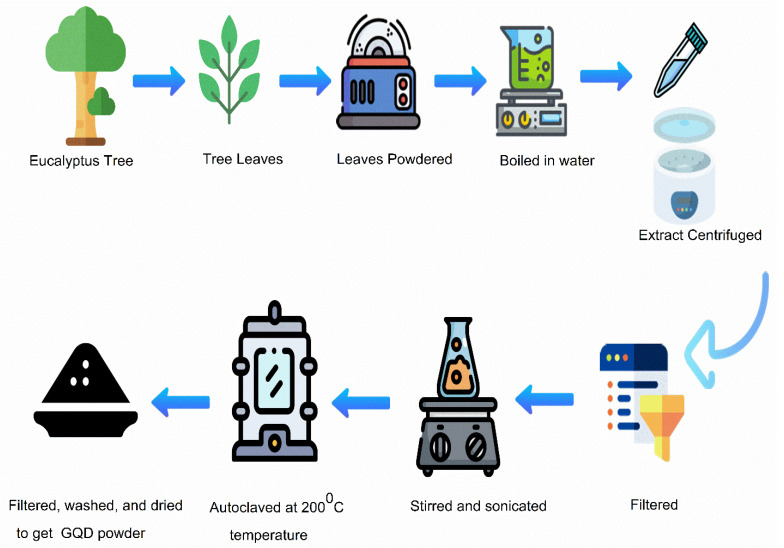
Schematic illustration of the preparation of GQDs from eucalyptus tree leaves.

The GQDs were prepared from leaf extract following the method described by Roy et al. [69]. Distilled water (200 mL) and the eucalyptus tree leaf powder (5 g) obtained by ball milling the eucalyptus tree leaves were boiled in water for 1 h at a temperature of 80 °C. Subsequently, the solution was centrifuged at a relative centrifuge force (RCF) of 10,000× *g* for ten min to remove any remaining solid residues. The supernatant obtained was then filtered using a 0.22-micron membrane to remove any solid residues present, before transferring it into a glass bottle. Then, the solution was properly stirred as well as sonicated at room temperature (25 °C) for 30 min. The solution was subsequently reacted in an autoclave for 13 h at 200 °C. The GQDs obtained were a mixture of black precipitates and a brown transparent suspension. After cooling down to room temperature (25 °C), this black precipitate was thoroughly discarded and then centrifuged at RCF 10,000× *g* for twenty minutes, the precipitate obtained was removed, and the supernatant that had been formed was collected and washed two times. The GQD solution was then filtered and dried properly. The schematic illustration of the preparation of the GQDs from eucalyptus tree leaves is presented in Figure 2.

### 2.3. Nanofiber Preparation Using SBS Technology

The production of a PES-based nanofiber membrane using SBS technology was completed as follows: initially, mixing PES pellets (25 wt.%) and N-methyl-2-pyrrolidone/toluene solvent mixture (2:1 wt.) and mixing well to obtain the PES solution. The air pressure of the system was kept at 2.0 bar, whereas the feeding rate of the polymer solution was 8 mL/h and 20 kV voltage. The PES solution was pumped across a 21-gauge needle, which was positioned within a concentric nozzle and at a working distance of 50 cm. The PES solution was pumped across the interior nozzle and a pressurized velocity gas was passed across the concentric exterior nozzle. The polymer concentration was 25 wt.% and the deposition time was 10 min for the nanofibers. The deposition was caried out for 10 min to achieve uniform distribution. The PES solution was fed across a concentric nozzle. As the solution came into contact with compressed air at the nozzle tip, it was stretched out with the shear effect developed by the air on the way to the collector, producing a nanofiber mat.

### 2.4. PES-Based NFM Post-Treatment by Heat Press

Heat-pressing post-treatment was performed on the PES-based NFM to enhance its morphology, as well as mechanical properties such as robustness and anti-wetting capabilities [70,71]. Heat-press post-treatment can also enhance the water permeation flux of the nanofiber membrane in water treatment applications and reduce the contact angle due to the nanofiber compaction [72]. The thickness of the PES-based NFM was in the range of 700–800 microns. Heat-press post-treatment works on the principle of hardening and increasing the mechanical properties of polymers by exposing the material to a temperature near to or above its glass transition temperature. The PES nanofiber supports, together with the polyester backing layer, were placed in the middle of a set of steel plates. The fabricated NFMs were heat-pressed at 150 °C for 10 min under 1.0 tons/m^2^ load. The steel plates were then removed from the heat press and left to cool.

### 2.5. Preparation of TFC/TFN Membranes

The PA layer used to prepare the TFC membrane was formed using the IP process on the surface of the nanofiber membrane support. The nanofiber membrane support was kept in distilled water for at least 12 h prior to the IP process. The control TFC-FO was synthesized using the IP process on the nanofiber membrane substrate. Initially, 2.0 wt.% MPD/distilled water aqueous solution was added to the membrane substrate for 120 s, and then the excess solution of MPD was eliminated from the membrane surface using a rubber roller. Subsequently, 0.15 wt.% TMC/hexane solution was poured on the membrane surface for 60 s. Then, all the synthesized TFC membranes were dried in air for 120 s and oven dried at 90 °C for 3 min, then stored in deionized water before further testing.

For the preparation of the TFN membranes, three different concentrations of GQDs (0.025 wt.%, 0.05 wt.%, and 0.075 wt.%) were mixed with MPD aqueous solution for the IP process. The preparation procedure for the TFN membranes was the same as the procedure for the preparation of the TFC membrane. The sample names are given in Table 1.

### 2.6. Characterization of the Synthesized GQDs and Membranes

The characterization of the nanomaterials was conducted to confirm the successful synthesis of the GQDs. The TFC and PA-TFN membranes were characterized to investigate the effects of incorporating nanomaterials on the membrane via the IP process.

The optical properties of the GQDs were studied using a UV-vis absorption spectrophotometer (Biochrom UV Spectrophotometer). The photoluminescence behavior of the GQDs was studied by employing a fluorescence spectrophotometer (FluoroMax-4 Spectrofluorometer-Horiba). GQDs can demonstrate tunable photoluminescence (PL) by means of the manipulation of edge functionality under different preparation conditions. The FTIR (760 Nicolet) was used to identify the inorganic and organic groups present in the sample. The morphologies of the GQDs were examined using TEM (HT 770, Hitachi, Tokyo, Japan). For Raman spectroscopy, the instrument used was the Thermo fisher scientific DXR Raman Microscope. It was used to analyze the impurities or defects present in graphitic carbon which affect the structure of native carbon. 

For membrane characterization, the SEM instrument used in this work was the Nova Nano SEM 450, and its voltage capacity ranged from 200 V to 30 kV. This SEM instrument was also equipped with an Energy Dispersive X-ray (EDX) unit. Characterization of the surface of membrane samples was carried out. To examine the surface roughness of the TFN membrane samples, a Veeco Metrology Nasoscope IV 3100 SPM was used. By using FTIR (760 Nicolet), it was possible to confirm the PA layer formation and the successful incorporation of GQDs into the TFC membranes. The water contact angle was measured using the contact angle system OCA (708381-T, LMS Scientific, Selangor, Malaysia).

### 2.7. Preparation of Solutions and Membrane Samples

In this study, 0.1 M NaCl in 3 L distilled water was used as the FS, and 1.5 M NaCl in 1 L distilled water was used as the DS in the experiments. The TFC and TFN membranes were cut to size as per the Sterlitech cell CF402F. To cut the membranes, the template cutter was used, and the cut membrane samples were loaded in the cell or stored for later use.

### 2.8. FO System Components

**Figure 3:**
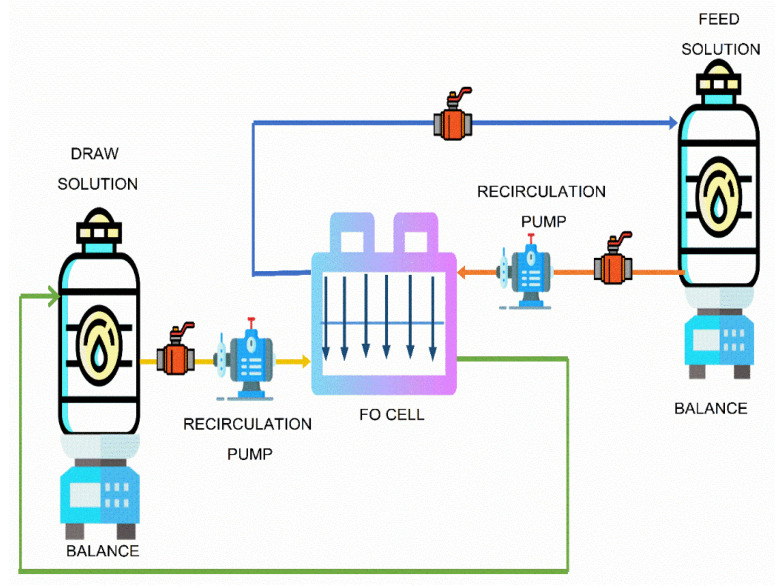
Diagrammatic representation of the FO System.

The main component of the FO system is the Sterlitech Cross flow CF042F cell (Figure 3) unit, which was purchased from Sterlitech Corporation. Two flow meters were used to monitor the flow on the feed and draw sides. In the FO system, the main performance was monitored by checking the rate of change of mass with respect to time and the change in conductivity.

### 2.9. FO System Operation

Precut membranes were conditioned in DI water for a minimum of 12 h and subsequently washed with DI water, before the membrane was placed in the cell. All FO experiments were operated in the AL-FS (FO) mode and AL-DS (PRO) mode. In the AL-FS mode, the active layer (AL) faced towards the FS, and the porous layer (PL) faced towards the DS side. In the AL-DS mode, the active layer (AL) faced towards the DS side, and the porous layer (PL) faced towards the FS side. At the beginning of the experiment, the FS and DS sides were connected to a backwash tank prefilled with 2 L of DI water on each side. The backwashing operation was carried out for a maximum of 30 min to clean the system and the membrane. After this, the main FO test was carried out with the FS and DS concentrations as 0.1 M and 1.5 M NaCl solutions, respectively. The temperature of both solutions was fixed at room temperature (25 °C) during all the FO runs. Flushing using DI water was performed after each run to overcome fouling and scale formation in the FO setup. In all the runs, only the membranes were changed while all the operational parameters were kept constant.

Both AL-DS and AL-FS modes were tested. *J_v_* (water flux) was calculated by measuring the weight change in FS:

(1) $$J_{v}=\frac{\Delta V_{feed}}{A_{m}\times \Delta t}=\frac{\Delta m_{feed}/\rho_{feed}}{A_{m}\times \Delta t}$$

where $\Delta V_{feed}$ and $\Delta m_{feed}$ are the volume change and weight change of the FS, respectively; $\rho_{feed}$ is the feed solution density; $A_{m}$ is the effective membrane area; and Δt is the measuring time interval. 

*Js* (solute flux) was calculated by determining the variation in salt content in FS on the basis of the conductivity measurements:

(2) $$J_{s}=\frac{\Delta \left(C_{t}V_{t}\right)}{A_{m}\cdot \Delta t}$$

where $C_{t}$ and $V_{t}$ are the salt concentration (g/L) and the volume of the FS (L) at the final stage of the experiment, respectively.

Specific reverse solute flux (SRSF) is calculated as *J_s_*/*J_v_*.

### 2.10. Effect of the Concentration of GQDs on the FO Performance

The effect GQDs loading (0.025 wt.%, 0.05 wt.%, and 0.075 wt.%) on the water flux, solute flux, SRSF, and salt rejection of the TFC membranes and the GQD incorporated TFN membranes were evaluated at AL-FS mode and AL-DS mode employing 0.1 M NaCl as the FS and 1.5 M NaCl as the DS.

### 2.11. Long-Term Performance Analysis of TFC/TFN Membranes

As the water flux of the developed membranes was very high, it was not possible to run the membranes continuously in a single run. Hence, eight runs were carried out continuously in the FO system without backwashing. Almost 108 liters of water were used during this long-term experiment and the experiments were carried out continuously for 7.5 h. Approximately 14 L FS of 0.1 M NaCl and 2 L DS of 1.5 M NaCl were used for each test.

The reversibility of the fouling layer, which is deposited on the polyamide selective layer, was examined by physical cleaning for 30 min with increased cross-flow velocity (two times the value employed during the long-term test). The cleaning effectiveness is shown in Equation (3).

(3) $$R\left(\%\right)=\frac{\left(J_{c}-J_{a}\right)}{\left(J_{b}-J_{a}\right)}$$

where *J_a_* is the water flux (L/m^2^h) after the fouling test; *J_b_* is the water flux (L/m^2^h) prior to fouling (fresh membrane) (LMH); and *J_c_* is the water flux (L/m^2^h) after physical cleaning.

### 2.12. Fouling Analysis of TFC/TFN Membranes

To examine the fouling resistance properties of the GQD-based TFC membranes, 100 mg/L HA was employed as a typical HA foulant. HA solution (100 ppm) was included in the feed solutions (0.1 M NaCl) during each fouling test and 1.5 M NaCl was utilized as the DS. For eliminating dilution of the DS, the baseline water flux data (*J_w_*) were achieved prior to each fouling test by employing a feed solution (0.1 M) with no HA foulant. Then, a predetermined concentration of HA solution was added to the feed solution. HA filtration was performed for a specific duration under the crossflow mode, and the steady flux with HA (*J_h_*) was noted. Subsequently, the used membranes were cleaned using deionized water. At the end of the test, the cleaned membrane’s steady water flux (*J_r_*) was examined using FS 0.1 M NaCl. The total flux recovery ratio (*FRR*) and the flux decline ratio (*FDR*) were used to evaluate the fouling resistance abilities of the TFC and nanocomposite membranes, which can be determined by means of Equations (4) and (5):

(4) $$FDR=\left[1-\frac{J_{h}}{J_{w}}\right]\times 100\%$$

(5) $$FRR=\left[\frac{J_{r}}{J_{w}}\right]\times 100\%$$

Normally, a higher *FRR* and a lower *FDR* represent the superior antifouling properties of the membranes.

## 3. Results and Discussion

### 3.1. Characterization of GQDs

#### 3.1.1. UV-Vis Spectrophotometry of GQDs

**Figure 4:**
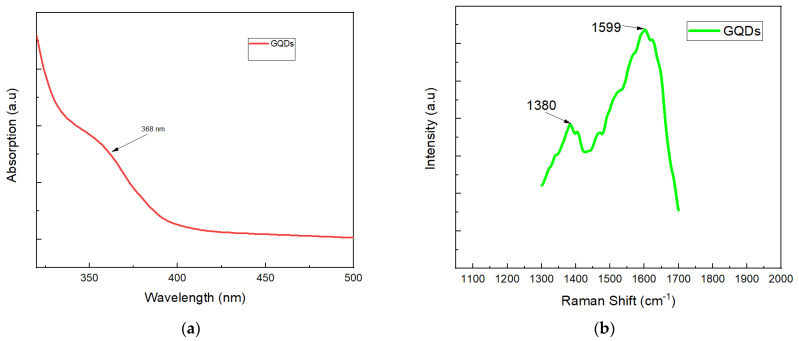

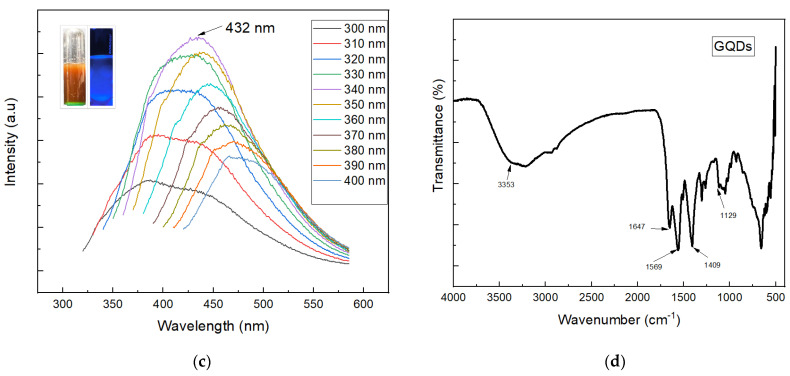
(**a**) UV-Vis Spectrum of GQDs (**b**) Raman spectrum of GQDs (**c**) photoluminescence spectra of the GQDs. Inset: photograph of the solution under daylight and UV light. (**d**) FTIR spectrum of GQDs.

The UV-Vis absorption spectrum for the GQD (Figure 4a) shows a strong background absorption at almost 368 nm, which is due to the π–π* transition of the aromatic sp^2^ domains. This is similar to characterization results observed by Roy et al. [69]. In the study by Roy et al. [69], GQDs were developed from the leaf extracts of Fenugreek and neem, and used for a white light-emitting diode (LED). The aqueous solutions of GQD showed a yellowish color and demonstrated blue luminescence under ultraviolet light, possibly because of the carbon particles with luminescent properties.

#### 3.1.2. Photoluminescence (PL) Spectroscopy of GQDs

The photoluminescence (PL) spectra of the prepared GQDs are presented in Figure 4c. From the examination of the photoluminescence spectra of the nanostructured material developed, it was noted that, at distinct excitation wavelengths from 300 to 400 nm, an enhancement in the emission intensity occurred until the maximum emission at 340 nm, and was subsequently reduced. The rise in the excitation wavelength resulted in a corresponding reduction in the photoluminescence emission intensity. The maximum PL intensity of the GQDs appeared at 432 nm. The fluorescence analyses of the GQDs reported in this study are consistent with those reported in several other studies [73,74]. The fluorescence emission mechanism of the GQDs is mainly due to the existence of an aromatic conjugated framework, a quantum size effect, oxygen-containing groups, and emission traps on the surface [75]. As is the case for other carbon-based nanomaterials with fluorescent properties, the excitation-dependent photoluminescence behavior of GQDs might arise from the optical selection of diversely sized GQDs and the surface defects in GQDs [76].

#### 3.1.3. TEM Analysis of GQDs

**Figure 5:**
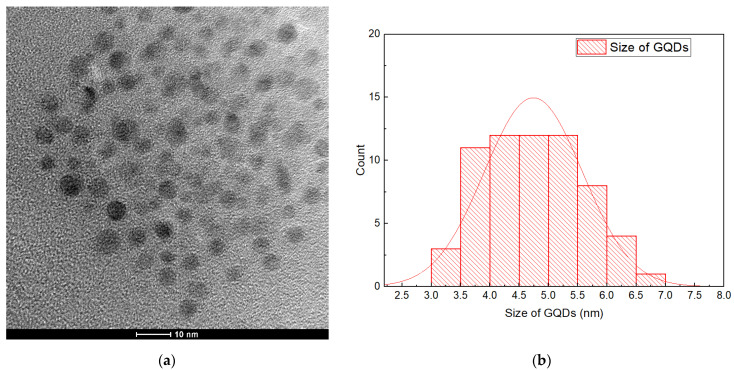
(**a**) TEM images of GQDs; (**b**) particle size distribution of GQDs.

The result of the TEM analysis of the prepared GQDs is shown in Figure 5a. The TEM image clearly confirms that the surface morphology of the GQDs was mainly spherical, and these nanoparticles were mono-dispersed. The particle sizes of various GQDs were examined by the histograms of the particle size distribution (Figure 5b) using Image J software. From the structural analysis, it was noted that the GQDs have a uniform particle size in the range of 3 nm to 7 nm, and were not aggregated. These GQDs have a narrow size distribution, with the major diameters ranging from 4.0 to 5.5 nm. This is in line with the results achieved by the study carried out by Kumawat et al. [77]. The development of the nanostructured GQDs might be due to the carbonization of the solution at the time of heat treatment in the autoclave. The carbonization degree of the material helps to control the size of the GQDs developed. We also noted that the resulting GQDs are mono-dispersed spherical particles. The emission properties of this nanomaterial are the result of its quantum size effect or the recombination of electrons and holes that takes place in the quantum-sized nanoparticles [78].

#### 3.1.4. Raman Analysis of GQD

Raman spectroscopic analysis is a technique used to examine the impurities or defects present in graphitic carbon, which impact the native carbon’s structure. The defects in the carbonic framework led to an increase in the defect or D-band in the Raman spectrum which was seen at ~1380 cm^−1^ in the GQDs (Figure 4b). This is an indication of a disorder developed in the crystalline sp^2^ carbon framework. Moreover, a graphitic G-band at ∼1599 cm^−1^ corresponded to the graphitic domains in the framework. The Raman spectrum confirmed that the D-bands at 1380 cm^−1^ developed from the disorder in sp^2^ hybridized carbon, and the G-bands at 1599 cm^−1^ corresponded to graphitic structures. The D band position in the Raman spectrum of the GQDs is not dependent on the quantum dot diameter, and the peak position of the G band varied with GQD size. The I_D_/I_G_ is an important measure of defect density, where the GQD edges represent defect sites in larger areas of graphene. The I_D_/I_G_ ratio of the amorphous quantum dots sample is high. A lower I_D_/I_G_ ratio indicates a higher degree of graphitization in the quantum dots sample. Here, the I_D_/I_G_ ratio of GQD is 0.528, suggesting that GQDs have a nano-crystalline graphite structure, which is almost identical to the results previously published [79].

#### 3.1.5. FTIR Analysis of GQD

The successful synthesis of GQDs from eucalyptus tree leaves using the hydrothermal treatment technique was confirmed by the result of the FTIR analysis. As shown in Figure 4d, the distinctive peak at 3353 cm^−1^ was due to the hydroxyl group stretching vibrations, and the carboxylate groups at 1647 cm^−1^ (asymmetric stretching vibrations of carbonyl bonds) proved the presence of –COOH functional groups in the GQDs [44,80]. The peak at 1409 cm^−1^ was due to the presence of C–H, whereas the peak at 1569 cm^−1^ was due to the aromatic ring’s C=C stretching vibrations, which confirms that the prepared GQDs consisted of an aromatic ring [81]. Moreover, the C–O (alkoxy) stretching peak was noted at 1129 cm^−1^. These FTIR results matched well with the results obtained by Xu et al. [41].

### 3.2. Membrane Characterization

#### 3.2.1. Mechanical Properties Analysis of Hot-Pressed NFM Support

In the current work, SBS-generated PES nanofibers were heat-press post-treated to improve the interconnectivity between the fibers and enhance the overall mechanical properties. The NFMs fabricated were heat-pressed at 150 °C for 10 min under 1.0 tons/m^2^ load. These values for temperature, time, and force were selected based on our previous studies performed on the nanofibers. Table 2 presents the mechanical properties of PES-based NFM supports before and after the hot-pressing post-treatment. The tensile strength increased from 5.470 MPa to 18.962 MPa, showing a 2.46-fold increase. Similarly, the Young’s modulus improved from 191.880 MPa to 535.370 MPa, demonstrating an increase of 1.79 times. This increasing trend confirmed that heat pressing under the specified conditions significantly enhanced the fiber strength. This increase in the mechanical properties is due to the fusion of the PES nanofibers on the upper and lower surfaces of the NFM. Furthermore, the higher values for the mechanical properties in the heat-pressed membranes can be explained by the inter-nanofiber interactions and the dense packing of the nanofibrous layers [82,83]. Thus, greater mechanical strength and lower thickness were achieved for the NFM substrate using this post-treatment method.

#### 3.2.2. AFM Analysis of TFC and GQD-Based TFN Membranes

**Figure 6:**
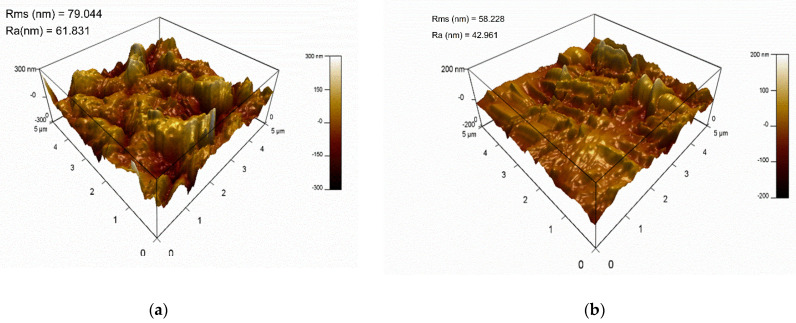
AFM pictures of (**a**) the TFC nanofiber membrane sample and (**b**) the TFN nanofiber membrane sample with 0.05−GQD/PA TFN.

To further check the surface morphologies of the TFC nanofiber membrane sample and the thin-film nanocomposite membrane samples, AFM analysis was performed, and the results are presented in Figure 6. The root-mean-squared roughness (R_rms_) and mean roughness (R_a_) are also included in Figure 6. It can be observed that the TFN membranes with GQDs are smoother relative to the TFC membrane. The roughness features of the membranes are reduced by the inclusion of GQDs, representing the GQD’s proper dispersion in the PA active layer. It was noted that the R_rms_ value was reduced from 79.044 to 58.228 by the inclusion of GQDs in the TFC membrane. The incorporation of GQDs could smooth the surface of the membrane [84] and also promote the effective development of the PA layer by means of the IP process [85]. Compared to the pristine TFC membranes, which had a rougher surface, the GQD-incorporated TFN membranes demonstrated smoother surfaces. Based on Seyedpour et al. [40], who incorporated citric-acid-derived GQDs into TFN membranes, there are two possible reasons for the developed membranes having smoother surfaces. First, the MPD diffusion normally produces a ridge-and-valley structure, while the horizontally aligned GQDs obstruct the MPD penetrating to the organic phase, which causes the development of a smooth surface. Second, the functional groups present on the GQD surface interacted with MPD and TMC monomers during the IP process, and these interactions impact the reaction rate of TMC and MPD [86]. 

#### 3.2.3. FTIR Analysis of TFC and GQD-Based TFN Membranes

**Figure 7:**
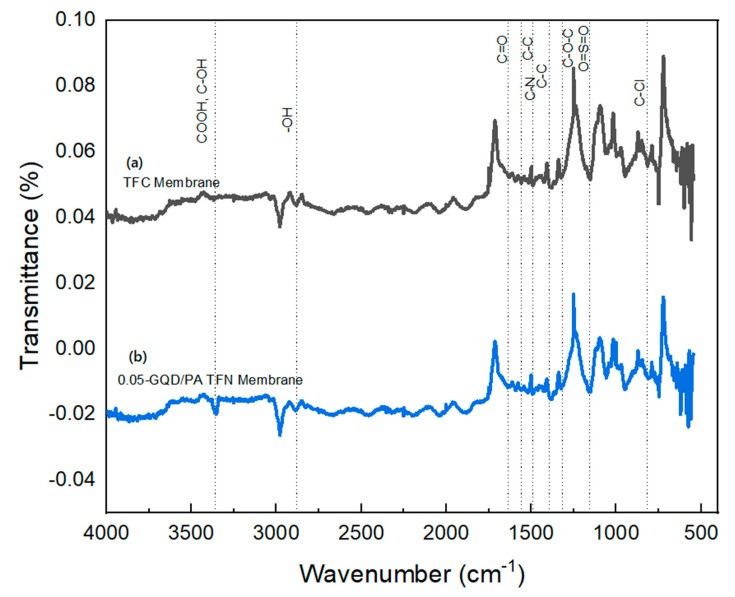
FTIR Images of (**a**) the TFC nanofiber membrane sample and (**b**) the TFN nanofiber membrane sample with 0.05−GQD/PA TFN.

The FTIR spectra of TFC and GQD-based nanocomposite membranes presented in Figure 7 confirm the development of the PA layer. The peaks observed at 1399 and 1486 cm^−1^ confirmed the presence of C–C in the aromatic ring [40,87]. The peaks developed at 1312 and 1152 cm^−1^ represent the asymmetric and symmetric O=S=O stretching vibrations of the PES substrate layer, respectively. The peak formed at 820 cm^−1^ corresponds to the C–Cl stretching vibrations of the unreacted acid chloride groups. The peak observed at approximately 1641 cm^−1^ arises due to the amide carbonyl stretching vibrations, while the band noted at almost 1561 cm^−1^ is mostly due to the interactions of C–N stretching, as well as the N–H in-plane bending vibrations [88,89]. Following the inclusion of the GQDs into the PA active layer, the intensity of the majority peaks was reduced significantly, and a few of them even vanished entirely with the presence of GQDs [88], demonstrating the interactions of the PA matrix and the GQDs [90]. Furthermore, the improved stretching vibrations of the carbonyl bond in GQD-incorporated membranes showed the development of the amide linkages by the interaction of the MPDs’ amine groups and carboxyl groups of GQDs [37]. Additionally, the peak seen at 1730 cm^−1^ for the GQD-based nanocomposite membranes is due to the stretching vibrations of carbonyl in the ester groups, developed by means of interactions between the carboxylic groups of the PA active layer and the functional groups of GQDs [88]. Relative to the pristine TFC membrane, the GQD-based nanocomposite membranes showed a greater intensity peak at approximately 3355 cm^−1^ which is due to the stretching vibrations of hydroxyl functional groups present in GQDs; this can improve the surface hydrophilic properties of the membranes, as noted from the CA results of the TFC and nanocomposite membranes [91]. The FTIR results obtained for the developed membranes in this work are in good agreement with the results reported by Seyedpour et al. [40].

#### 3.2.4. Contact Angle (CA) Analysis of TFC and TFN Membranes

**Figure 8:**
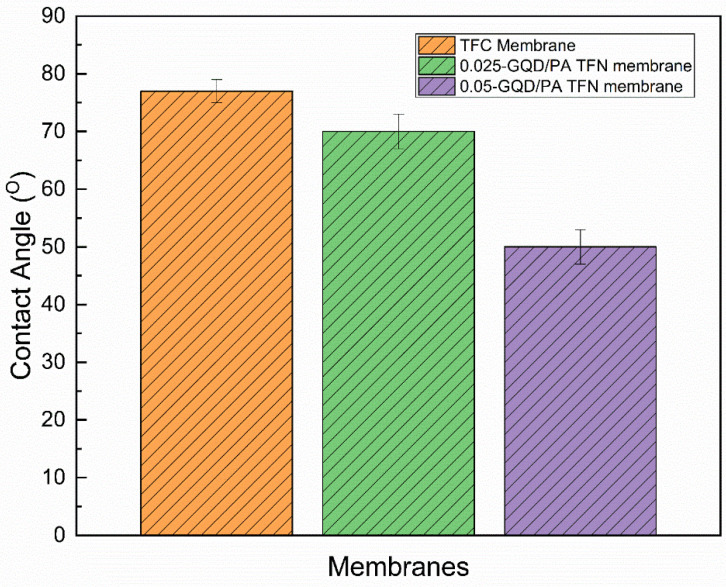
Contact angle analysis of the TFC nanofiber membrane sample and the TFN nanofiber membrane sample with 0.025-GQD/PA TFN and 0.05-GQD/PA TFN.

Figure 8 shows the CA values of the TFC and GQD-based nanocomposite membranes. The inclusion of GQDs substantially enhanced the hydrophilic properties of the PA active layer, reducing the water contact angle from 77° (for the TFC membrane) to 70° (for 0.025-GQD/PA TFN) and 50° (for 0.05-GQD/PA TFN). From the FTIR results for the TFN membranes, it was noted that there were stretching vibrations of hydroxyl functional groups present in GQDs, and this resulted in enhanced surface hydrophilicity [91]. This considerable improvement in the hydrophilic properties of the GQD-based nanocomposite membranes could be due to the presence of functional groups with plentiful oxygen-comprising groups on the exterior of GQDs [89]. This increase in surface hydrophilicity can reduce the adhesion of foulants, thus enhancing the fouling resistance [92,93]. The contact angle analysis matched well with the results obtained in the research work carried out by Seyedpour et al. [40].

Furthermore, it can be noted that surface roughness plays a significant role in surface wettability. With the addition of 0.05 wt.% GQD, the surface roughness value was reduced and the membrane hydrophilicity increased. When a water droplet entirely wets a rough surface on which it is placed, the effect of surface roughness on CA is demonstrated by the Wenzel equation ($\cos\theta_{W}=r\;\cos\theta_{Y}\;$, where $\theta_{W}$ is the observed CA on a rough surface, r is the roughness ratio, and $\theta_{Y}$ is the CA on a rough surface) [94]. Due to the fact that the roughness ratio compares the rough surface’s true surface area with the surface area of a comparably sized smooth surface, the roughness ratio will always be greater than one. Wenzel’s relation thus confirmed that surface roughness would reduce the CA value for a droplet on a hydrophilic surface and increase the CA for a droplet on a hydrophobic surface. In the current study, with the incorporation of GQDs, the surface became more hydrophilic (the CA value was reduced), meaning that it showed a decreased surface roughness value, according to the Wenzel equation.

#### 3.2.5. SEM Analysis of TFC Membranes

**Figure 9:**
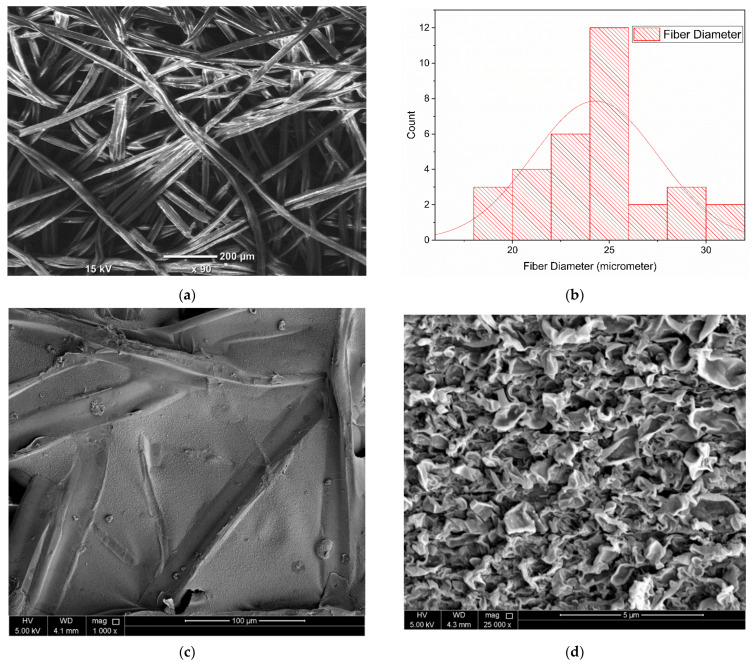

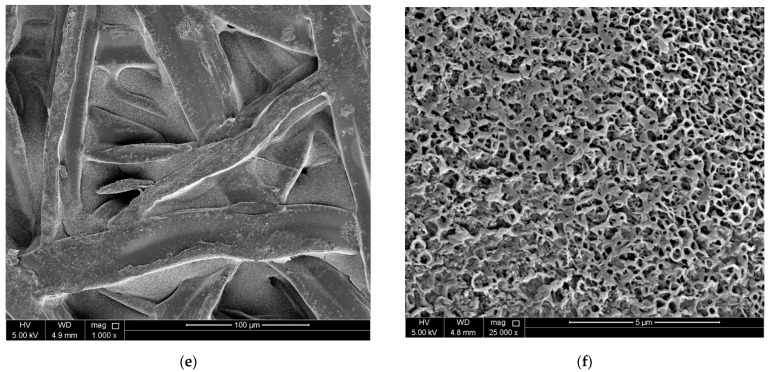
SEM Images of (**a**) nanofiber membrane, (**b**) fiber diameter distribution in the nanofiber membrane, (**c**) top surface view of the pristine TFC at 1000× magnification, (**d**) top surface view of the pristine TFC at 25,000× magnification, (**e**) top surface view of 0.05-GQD/PA TFN at 1000× magnification, (**f**) top surface view of 0.05-GQD/PA TFN at 25,000× magnification.

Representative SEM images of the surface morphology of the nanofiber supports and TFC and nanocomposite membranes are shown in Figure 9. Figure 9a presents the SEM image of the nanofiber membrane, and the uniform fiber network can clearly be observed from the figure. From the fiber diameter analysis (Figure 9b), it can be noted that the majority of the fibers have fiber diameters in the range of 20 to 25 μm. The PA layer of the TFC membrane was deposited on top of the PES nanofiber support through the IP process only after heat-press post-treatment of the membranes. Figure 9c presents the top view of pristine TFC membrane at 1000× magnification. It is important to note that the patterns and shapes of the SBS-generated nanofibers were evidently stamped beneath the PA layer, and this is normally not noticed in the pristine TFC membranes fabricated using the phase inversion technique [95]. A similar PA layer structure has been observed in previous works in which PA layer was developed on top of electrospun NFM supports [96,97,98]. The NFM substrate has superior porosity, high surface roughness, and larger interstitial pore frameworks, which possibly generate the development of this specific kind of PA selective layer. Moreover, Figure 9d shows the top view of the pristine TFC at 25,000× magnification. Standard ridge-and-valley frameworks were noted on the surface of the TFC membrane, which indicates the effective formation of the PA layer. Figure 9e shows the top view of the 0.05-GQD/PA TFN membrane at 1000× magnification, while Figure 9f presents a zoomed-in image of the same membrane at 25,000× magnification. It can be noted that the GQDs are unnoticeable on the surface of the PA selective layer, confirming that these materials are properly incorporated within the PA layer [99].

#### 3.2.6. EDX Analysis of TFC Membranes

**Figure 10:**
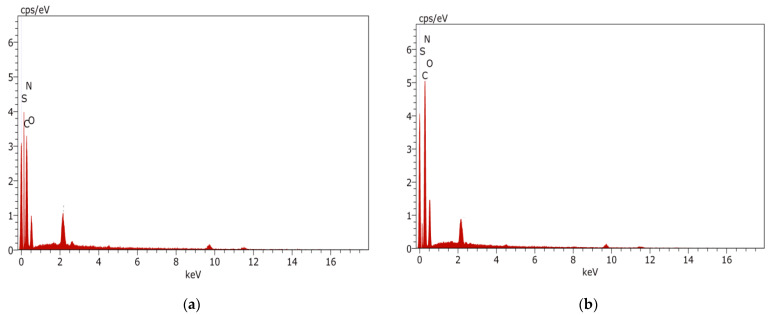
(**a**) EDX analysis of the TFC membranes, (**b**) EDX analysis of the 0.05-GQD/PA TFN membrane.

EDX carried out during the SEM analysis confirmed the presence of carbon, nitrogen, and oxygen. As shown in Figure 10 and Table 3, carbon is the major element present in the TFC and 0.05-GQD/PA TFN membranes. It can be noted that, with the incorporation of GQDs into the membrane, the carbon content increased from 57.47% to 62.07%. This is due to the high carbon concentration of GQDs [100]. This clearly confirmed the presence of GQDs in the TFN membrane. Regarding nitrogen, it was found that the atomic% of individual nitrogen atoms was reduced from 14.85% to 8.46% by the addition of GQDs to the membrane. This is due to the fact that the amine functional groups present in the MPD monomer might undergo a reaction with GQDs, thereby reducing the nitrogen atomic percentage. As oxygen element present in the TFC membrane does not have any interactions with GQDs, its atomic percentage remained almost the same. Thus, the EDX analysis confirmed the presence of GQDs in the TFN membrane. 

### 3.3. Performance Analysis of TFC/TFN Membranes

The effects of GQD loading on the performance of the FO membrane (water flux, solute flux, SRSF, and salt rejection), the long-term performance analysis of the developed membranes, and its organic fouling resistance were investigated. We refer the reader to the Supplementary Materials for the effect of feed side and draw side flow rates on the water and salt flux of TFC membranes. Table S1 presents the effect of three different feed side and draw side flow rates on water and salt flux of TFC membranes. Figure S1 shows the impact of FS and DS flow rates on water and salt flux of TFC membranes.

#### 3.3.1. Effect of GQD Loading on the FO Performance

**Figure 11:**
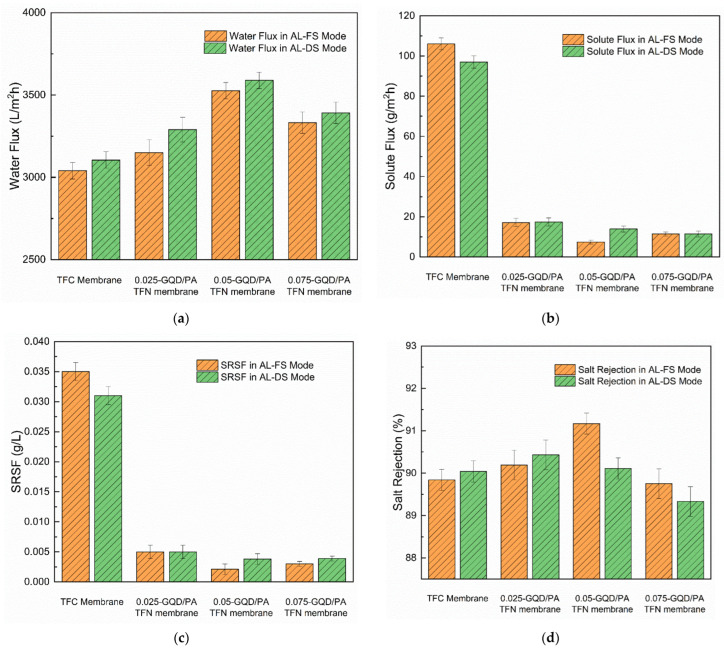
The impact of different concentrations of GQDs on the performance of TFC and TFN membranes. (**a**) Water flux of the developed membranes in AL-FS and AL-DS modes, (**b**) solute flux of the developed membranes in AL-FS and AL-DS modes, (**c**) specific reverse solute flux of the developed membranes in AL-FS and AL-DS modes, (**d**) salt rejection of the developed membranes in AL-FS and AL-DS modes.

The effect of GQDs loading on the water flux, solute flux, SRSF, and salt rejection of the TFC membranes and the GQD-incorporated thin-film nanocomposite membranes were assessed at the AL-FS mode and the AL-DS mode using 0.1 M NaCl as the FS and 1.5 M NaCl as the DS. The results are presented in Figure 11.

From the FO performance analysis, it was noted that the TFC and TFN membranes showed superior performance by demonstrating ultrafast water flux in the range of 3000 L/m^2^h. The reason for the ultra-fast water flux is explained in the following section. The nano porous structure of membrane is the most important factor, as it controls the water flux and salt rejection. In a study by Medeiros et al. [101] it was stated that SBS-produced nanofibers have more open pore structure than those produced by electrospinning. The overall porosity (77–95%) and pore size (8–17 μm) of the SBS scaffolds are greater than those of scaffolds produced by electrospinning with similar polymers (67% and 3 μm, respectively) [102]. As per our pore size analysis on the SEM images of the nanofiber substrate image, carried out using the ImageJ software, it was noted that the pore size is in the range of 11 μm. Moreover, the membrane fabricated with spin blown technology shows an array of highly regular/irregular sub-nano meter pores and channels and a large volume of free space, which increases the water permeability. Zheng et al. [103] carried out a desalination experiment using just the PA layer and confirmed that the PA membrane with no support layer results in the support having porosity of 100%, which leads to reduced mass transfer resistance as well as decreased internal concentration polarization effects. Hence, the nanofibers fabricated using the SBS technique show increased porosity, which is inclined towards 100% porosity, thereby providing extremely high water flux. Moreover, in real cases, nonideal factors such as irregular wooing (long zig-zag pathways) and pinhole-like pathways (short pathways) greatly impact transport pathways and this structure is possibly present in the SBS-produced nanofiber membranes. Since short pathways are created, water flow prefers to take shortcuts to reduce the travel resistance, hence the high-water flux. Moreover, the high surface volume ratio of the SBS-produced fibers is another major reason for the ultrahigh water flux. It can be noted that the water molecular diameter is 0.28 nm, and very small relative to the nanofiber pore size [104]. The water molecules have good cohesion properties, hence the water molecules are attracted to themselves, which allows water to be a very “sticky” liquid. This cohesion property is due to hydrogen bonding, i.e., the electrostatic attractions caused by the difference in charge between slightly positive hydrogen ions and slightly negative OH ions. For water, the hydrogen bonds are formed between neighboring hydrogen and oxygen atoms of adjacent water molecules. In the case of small pore-sized membranes, the accumulated water molecules face a stretch when passing through the pores; as such, the hydrogen bonds are broken by mechanical means to enable its passage through the small-sized pores. However, in the case of this SBS-produced nanofiber membrane, due to its larger pore size as compared to other reported membranes, the passage of bulk/accumulated hydrated water molecules is permitted, instead of the passage of individual water molecules; this is a major reason for the ultrahigh water flux.

All the GQD-based nanocomposite membranes exhibited an increased water flux, lower solute flux, and lower SRSF, as compared to the TFC membrane. In AL-FS mode, the water flux of the TFC membrane was noted to be 3040 L/m^2^ h. With the incorporation of the GQDs, the water flux of the membranes first increased with 0.025 wt.% (3150 L/m^2^ h) and 0.05 wt.% (3526 L/m^2^ h), and then decreased with 0.075 wt.% GQD content (3332 L/m^2^ h). Thus, an increase of 3.5%, 16%, and 9.5% in water flux was accomplished with the incorporation of 0.025 wt.%, 0.05 wt.%, and 0.075 wt.% GQDs, respectively, as compared to the TFC membrane. This increase in the water flux can be primarily explained by the water channels developed between the PA selective layer and the GQDs, which provided additional channels for the penetration of water. It may offer additional nano-channels for the transportation of water molecules by the interfacial gap between the PA structure and the GQD nanosheets [40]. Furthermore, the extra driving force derived from the hydrogen bond interactions between the hydroxyl/carboxyl groups of the GQDs and water molecules accelerates water molecules for penetrating within the water channels. The above-mentioned notes indicate that the inclusion of the GQDs in the PA layer enhances the membrane surface’s hydrophilicity by providing a faster water molecule transfer through the membrane [40,89]. These results were confirmed by the contact angle analysis results. Nevertheless, the water flux was reduced with high loads of GQDs, as noted in the 0.075 wt.% GQD-incorporated TFN membrane. This could be due to the GQD agglomeration, which does not offer so many nanocorridors, and can even cause pore blocking on the substrate surface. At 0.075 wt.% GQD incorporation, an uneven distribution of GQDs occurs inside the PA layer, which has less hydrophilicity. Extreme GQD accumulation reduces the effective surface area of the nanoparticles, thus lowering the number of hydrophilic groups of GQDs exposed on the PA layer surface. Furthermore, GQD aggregates can hinder water transport by blocking certain substrate pores. Excessive GQDs at higher concentrations can also cause very circuitous and elongated transfer paths for the transport of water molecules within the PA selective layer. Consequently, the hydraulic resistance is intensified, and the water flux reduced. The steric hindrance effect of GQD aggregates inhibits the IP process by stopping MPD from immediately undergoing diffusion into the TMC organic phase and slow down the ridge development in PA layer. Therefore, the PA layer develops around the GQD aggregates and makes non-selective areas that can permit DS ions to pass across the membrane to the FS side. Moreover, all the TFN membranes showed greater water fluxes in AL-DS mode than those in AL-FS mode. This is due to the fact that the internal concentration polarization impact in the AL-DS membrane configuration is concentrative, which is weaker than the alternate mode [105]. The 0.05 wt.% GQD incorporated membranes showed maximum flux in both AL-FS (3526 L/m^2^ h) and AL-DS (3589 L/m^2^ h) modes. There was an improvement of almost 16% in water flux in the AL-FS mode and a 13.5% increase in the AL-DS mode. 

Conversely, regarding the solute flux of the GQD membranes, the solute flux decreased with the incorporation of GQD content. The 0.05 wt.% GQD-incorporated membranes showed a minimum solute flux of almost 93% in AL-FS mode and 85.6% decrease in AL-DS mode. In 0.05 wt.%-GQD membrane AL-FS mode operation, the solute flux reached to a decreased value of 7.46 g/m^2^h. However, the 0.075 wt.%-GQD membrane demonstrated a small increase in the solute flux value as compared to the 0.05 wt.% GQD-incorporated membrane. Furthermore, the SRSF (*J*_s_/*J*_w_) is a significant parameter that reflects the performance of a membrane with respect to productivity and selectivity in the FO process. In general, a membrane with a lower *J*_s_/*J*_w_ value can contribute adequate selectivity in rejecting salt as compared to water. In the current work, 0.05 wt.% GQD-incorporated membranes had decreased values of *J*_s_/*J*_w_ (0.0038 g/L in AL-DS mode and 0.0021 g/L in AL-FS mode) and demonstrated better performance as compared to some lab-prepared FO membranes [106,107,108]. With the increasing GQD load, the SRSF was initially reduced; it reached the minimal value at 0.05 wt.% GQD concentration and was then marginally enhanced, which confirmed the reverse trend of the salt rejection of the TFC membranes. Similar FO performance was noted in a study carried out by Xu et al. [41]. The *J*_s_/*J*_w_ of 0.05 wt.% GQD-incorporated membranes showed a significant decrease compared to that of TFC membrane. 

Furthermore, it was observed that the salt rejection of the 0.025 wt.% GQD membrane and the 0.05 wt.% GQD membrane was slightly increased relative to the pristine TFC-0 membrane. However, the 0.075 wt.% GQD membrane showed similar salt rejection to the TFC membrane. The additional inclusion of GQDs did not contribute to an increase in the salt rejection due to the aggregation as well as the uneven distribution of GQDs within the PA layer [40,89]. Therefore, it can be noted that the water flux as well as the salt rejection of GQD-based TFN membranes may be improved by incorporating a suitable amount of GQDs into the PA selective layer, which is always a trade-off in TFN membranes. The incorporation of an adequate wt.% of GQDs into the PA layer can improve the surface hydrophilicity of the membrane by contributing faster water molecule transfer through the membrane. The 0.05-GQD/PA TFN showed increased water flux, decreased SRSF, reduced solute flux, and increased salt rejection, as compared to the TFC membrane.

When the GQDs were added to the MPD solution, these nanomaterials could interact with MPD and TMC monomers during the IP process, permitting the proper inclusion of the GQDs into the PA selective layer. Apart from the hydrogen bonds, the amine functional groups present in the MPD monomer can undergo a reaction with GQDs and develop new amide bonds during the ultrasonication of the MPD solution containing the GQDs. Moreover, the formation of anhydride and ester linkages is expected, owing to the interaction between the functional groups of GQDs and the acid chloride groups of TMC. The unreacted acid chloride groups of TMC might interact with the carboxyl groups of the GQDs during the IP process. The hydrogen bonding can be developed by the interactions between the functional groups of the GQDs and the primary as well as secondary amines. Furthermore, covalent bonding between the carboxyl groups in the linear fraction of the PA layer and GQDs might be developed by means of condensation reactions. Moreover, the steric hindrance of the GQDs lessens the penetration of MPD and slows down the development of the selective PA layer [109]. The mass transfer resistance is significantly reduced with the reduction in the thickness of the PA layer, resulting in an increase in the water flux [86]. Nevertheless, by employing an increased wt.% of GQDs, the total thickness of the PA layer of the nanocomposite membranes was enhanced, and a denser layer was developed, relative to that of the TFC membranes. However, it must be noted that the higher concentration of GQDs in the membrane leads to GQD leakage from the selective layer and severe agglomerations, as well as a thicker active layer [40].

#### 3.3.2. Long-Term Performance Analysis of TFC/TFN Membranes

**Figure 12:**
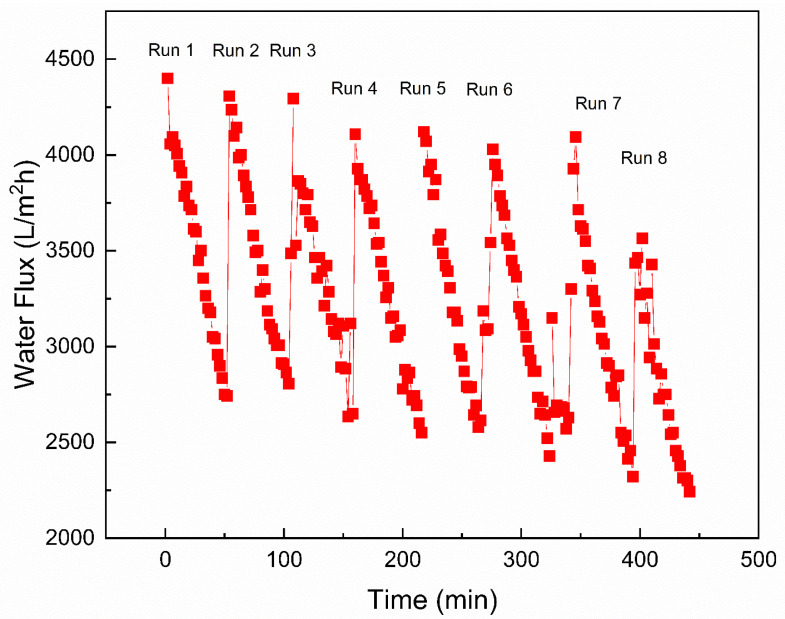
Water flux (L/m^2^ h) of the 0.05-GQD/PA TFN membranes during long-term performance analysis—eight runs, i.e., 7.5 h in total.

**Figure 13:**
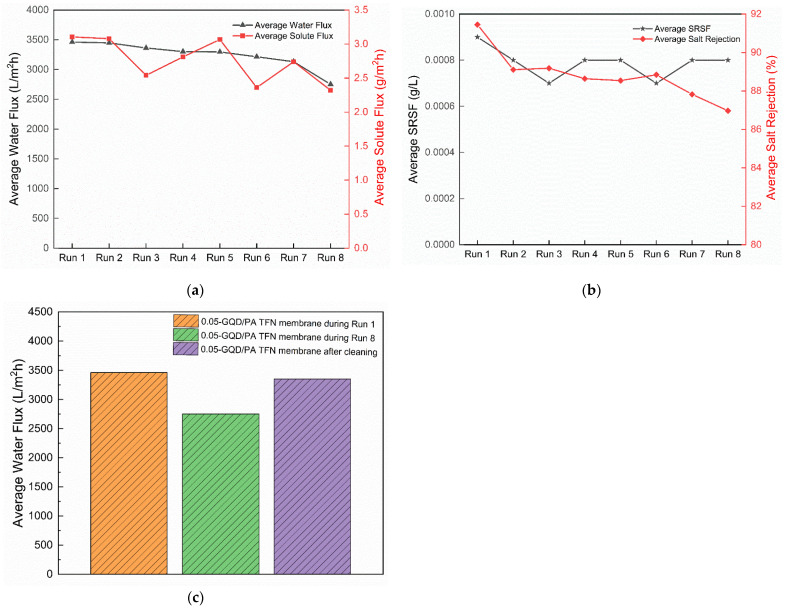
(**a**) Average water flux (L/m^2^ h) and average solute flux (g/m^2^ h) of the 0.05-GQD/PA TFN membrane during long-term performance analysis—8 runs (**b**) Average SRSF (g/L) and Average salt rejection (%) of the 0.05-GQD/PA TFN membranes during long-term performance analysis—8 runs (**c**) Cleaning efficiency after long term FO Testing with 0.05-GQD/PA TFN membrane.

Figure 12 demonstrates the long-term water flux performance analysis of the 0.05-GQD/PA TFN membrane in an FO process. High water flux was achieved during the start of the FO operation and later it reduced with time. The FO flux reduction was influenced by the dilution of the DS and the FS concentration [110]. The behavior of the flux decrease with time was demonstrated by Ali et al. [111] and Zhao et al. [112]. Zhao et al. [112] confirmed that membrane fouling and internal concentration polarization could decrease the osmotic water flux and enhance mass transfer resistance. This is because the feed solution turned out to be very concentrated due to the water permeation from the feed solution to draw solution and reverse salt diffusion from the draw solution to feed solution. As illustrated in Figure 13a, the average water flux for the first run was 3462 L/m^2^ h. It decreased to 3447 L/m^2^h during the second run and to 2752 L/m^2^ h after the eighth run.

As shown in Figure 13a, it was also noted that the solute flux of the 0.05-GQD/PA TFN membrane decreased gradually with time. The solute flux during the first run was 3.105 g/m^2^ h, whereas it was reduced to 2.32 g/m^2^ h during the eighth run. Similarly, a small reduction was observed for the SRSF value, which was 0.0009 g/L after the first run and reached 0.0008 g/L after the eighth run (Figure 13b). The behavior of RSF with time was slightly influenced by the decrease in water flux. Figure 13b also demonstrates the salt rejection of the 0.05-GQD/PA TFN membrane as a function of FO permeation time during the long-term experiment. It was found that the salt rejection declined from about 91.446% to 86.97% after 7.5 h.

Following cleaning for one hour, it was noted that the water flux recovered to 84% of the original flux value. The development of the boundary layer is less compact, as FO is not a pressure-driven process. Thus, the loosely deposited foulants could be obviously separated by a high crossflow velocity rate. Despite the decrease, the water flux recovery of 84% was achieved after the fouling–cleaning tests, which could be due to the enhanced hydrophilicity of the membranes. Figure 13c presents the cleaning efficiency after long-term FO testing with GQD membranes. It is noted that the FO performance is almost reversible after proper cleaning of the developed membranes, even after long-term experiments. 

#### 3.3.3. Organic Fouling Resistance Analysis of the Developed TFC/TFN Membranes

**Figure 14:**
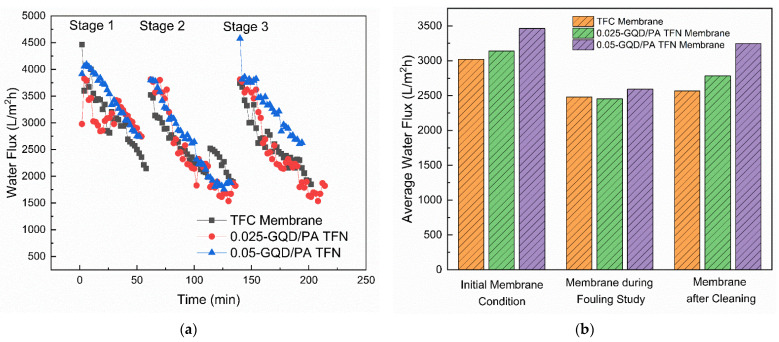
(**a**) Fouling study of the TFC and GQD-based membranes. Stage 1: initial flux (FS: 0.1 M NaCl). Stage 2: fouling study (FS: 0.1 M NaCl+ HA solution). Stage 3: flux after cleaning (FS: 0.1 M NaCl). (**b**) Average water flux of the TFC and GQD-based membranes during the fouling study and cleaning.

**Figure 15:**
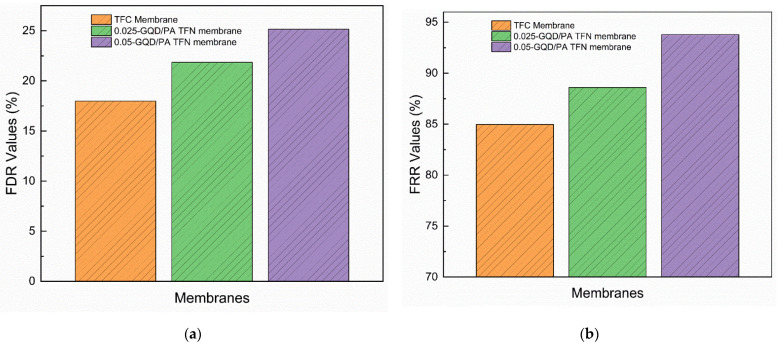
(**a**) Total flux decline ratio (FDR) values of TFC and GQD-based membranes during the fouling study. (**b**) Flux recovery ratio (FRR) values of TFC and GQD-based membranes during the fouling study.

The fouling resistance ability of the GQD-based TFN membranes was examined using 100 mg/L HA. The HA was employed as a representative foulant. HA solution (100 ppm) was added to the feed solutions for each fouling experiment. Additionally, 1.5 M NaCl was used as the DS. The fouling behavior of the TFC membrane, the 0.025 wt.% GQD membrane, and the 0.05 wt.% GQD membrane are shown in Figure 14a. These three membranes were selected to determine the effect of GQD concentration on the fouling resistance ability of the membrane. As the 0.075-GQD/PA TFN membrane showed a decrease in FO performance as compared to the 0.05-GQD/PA TFN membrane, this membrane was not used in the organic fouling resistance study. In the first stage, the original water flux of the three membranes was established using 0.1 M NaCl as the feed solution. In the second stage, the HA solution was added to the feed solution. Subsequently, it was noted that all the TFC and GQD-incorporated membranes suffered a reduction in flux. As shown in Figure 15a, the FDR values of the TFC, 0.025-GQD/PA TFN, and 0.05-GQD/PA TFN membranes were 17.98%, 21.8%, and 25.1%, respectively. These results prove that the HA fouling resistance of the TFC membranes was enhanced by the inclusion of the GQDs in the PA selective layer. Additionally, the rise in the FDR values showed that the antifouling properties improved as the GQD concentration increased. In the second stage, the FO process was carried out for 72 min, and, later, the membranes were physically cleaned with deionized water. Figure 14b shows the average water flux of the TFC and GQD-based membranes during the fouling study and cleaning. It was confirmed that the fouling is almost reversible, and the 0.05-GQD/PA TFN membrane almost retained the original water flux value.

In the third stage, the cleaned TFC and TFN membranes were filtrated using 0.1 M NaCl as the feed solution, and the flux was noted (Figure 14a). The FRR values of the TFC membrane, the 0.025-GQD/PA TFN membrane, and the 0.05-GQD/PA TFN membrane were 84.96%, 88.59%, and 93.78%, respectively (Figure 15b). Relative to the pristine TFC membrane, the higher FRR values of the GQD-TFN membranes revealed a higher cleaning effectiveness with an appropriate concentration of GQDs. The FRR and FDR results prove that the GQD-incorporated TFN membranes demonstrate better performance than the pristine TFC membrane in both HA antifouling properties and cleaning effectiveness. The increased hydrophilicity was mainly responsible for the increase in the antifouling performance of the GQD-TFN membranes. With respect to hydrophilicity, the inclusion of hydrophilic GQDs rendered the surfaces of the GQD-TFN membranes extremely hydrophilic [113]. The membrane surface’s hydrophilic properties might reinforce its binding capability to water molecules on the surface and inside the PA selective layer, developing a hydrated layer for resisting the foulants [114]. Therefore, the greater hydrophilic properties of the GQD-TFN membranes favorably assisted in their strong antifouling properties. Considering the FO performance study (water flux, solute flux, SRSF, salt rejection), the long-term performance analysis, and the organic fouling resistance study, the 0.05-GQD/PA TFN membrane was determined to be the best membrane among the membranes developed in this study. 

## 4. Conclusions

In summary, GQDs prepared from eucalyptus tree leaves were used in the selective layer of TFC membranes for FO application. This preparation process involves a simple and green one-pot hydrothermal technique employing DI water as a solvent for the first time. The process involves no additional organic solvent, passivizing, or reducing agent. Moreover, the nanofiber support of the TFC membranes was prepared using an advanced SBS method. This is the first study that reports the fouling resistance of the SBS-nanofiber-supported TFC membranes. In this study, the characterization data of the GQDs, TFC, and GQD-based TFN membranes were presented. The UV-Vis, PL, and FTIR analyses of the developed GQDs confirmed the successful formation of GQDs. The TEM analysis confirmed that the GQDs have particle sizes in the range of 3 to 7 nm. The TFN membranes showed better surface smoothness and hydrophilicity compared to the TFC membranes. The AFM, FTIR, CA, and EDX analyses confirmed the successful incorporation of the GQDs into the PA matrix of the TFC membrane. The effect of GQD loading on the FO membrane’s performance (water flux, solute flux, SRSF, and salt rejection), the long-term performance analysis, and the fouling resistance of the TFN membranes was investigated. It was noted that the overall FO separation performance of the TFC membranes was improved with the addition of 0.05 wt.% GQDs. All of the TFN membranes showed greater water flux values in the AL-DS mode relative to those in the AL-FS mode. This work confirms that the green-synthesized GQD-incorporated TFC membranes, with SBS nanofiber support, possess outstanding FO performance with respect to water flux, selectivity, and antifouling performance. The fouling of the GQD-added TFN membranes was almost reversible, demonstrating its great potential for FO applications.

## Figures and Tables

**Table 1:** Naming of the different developed membranes.

GQD Amount	Membrane Name
0.025 wt.%	0.025-GQD/PA TFN
0.050 wt.%	0.05-GQD/PA TFN
0.075 wt.%	0.075-GQD/PA TFN

**Table 2:** Mechanical properties of PES-based NFM supports before and after hot-pressing post-treatment.

	Tensile Strength (Mpa)	Young’s Modulus (Mpa)	Thickness (Microns)
NFM sample before hot-pressing	5.470	191.880	730
NFM sample after hot-pressing	18.962	535.370	388

**Table 3:** Elemental compositional analysis of the TFC and 0.05-GQD/PA TFN membranes.

Elements	Atom. C in TFC Membrane [at. %]	Atom. C in 0.05-GQD/PA TFN Membrane [at. %]
Carbon (C)	57.47	62.07
Nitrogen (N)	14.85	8.46
Oxygen (O)	27.39	29.35
Sulfur (S)	0.29	0.11

## Supplementary Materials

The following are available online at https://www.mdpi.com/article/10.3390/nano12193519/s1, Figure S1: Impact of FS and DS flow rates on water and salt flux of TFC membranes. Table S1: Effect of three different feed side and draw side flow rates on water and salt flux of TFC membranes.
