# Supplementary material for: Improved Forward Osmosis Performance of Thin Film Composite Membranes with Graphene Quantum Dots Derived from Eucalyptus Tree Leaves
Source: Nanomaterials (Basel). 2022 Oct 8;12(19):3519. doi: 10.3390/nano12193519 (PMC9565292; doi:10.3390/nano12193519)
Supplement: Supplementary file 1 [file nanomaterials-12-03519-s001.zip › nanomaterials-1959387-Supplementary Materials .pdf]

Supplementary Materials

Article

Improved Forward Osmosis Performance of Thin Film Composite Membranes with Graphene Quantum Dots Derived from Eucalyptus Tree Leaves

Haleema Saleem [1], Asif Saud [1], Nazmin Munira [1], Pei Sean Goh [2], Ahmad Fauzi Ismail [2], Hammadur Rahman Siddiqui [1] and Syed Javaid Zaidi [1,*]

[1] UNESCO Chair on Desalination and Water Treatment, Center for Advanced Materials, Qatar University, Doha P.O. Box 2713, Qatar
[2] Advanced Membrane Technology Research Centre, School of Chemical and Energy Engineering, Universiti Teknologi Malaysia, Johor Bahru 81310, Malaysia
* Correspondence: szaidi@qu.edu.qa or smjavaidzaidi@gmail.com; Tel.: +0097444037723.

3.3.1. Effect of feed side and draw side flow rates on water and salt flux of TFC membranes

Table S1. Effect of three different feed side and draw side flow rates on water and salt flux of TFC membranes.

Flow rates	Feed side (CCM)	Draw side (CCM)	Water flux (L/m^2h)	Solute Flux (g/m^2h)
F1	600	300	1287	2920
F2	1000	400	3040	106.44
F3	1500	600	4873	1157.2

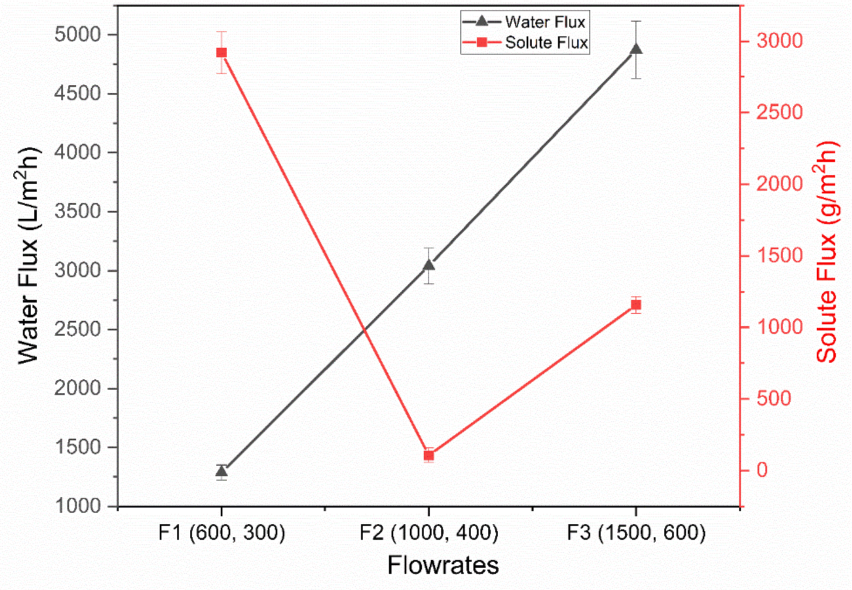

Figure S1. Impact of FS and DS flow rates on water and salt flux of TFC membranes.

The impact of increasing feed and draw solution flow rates on the membrane performance was studied using 0.1 M NaCl and 1.5 M NaCl as feed and draw solution, respectively. The FO tests were conducted using the TFC membrane in AL-DS orientation at room temperature. This test was carried out to determine the best flow rate on the feed side and draw side, which could offer high water flux and low solute flux. For the feed side, the flow rates varied from 600 to 1500 cubic centimeters per minute (CCM), whereas for the draw side, the flow rates varied from 300 to 600 CCM. It can be observed from Fig. S1 and Table S1 that the water flux increased by almost 54% from 1287 LMH to 3040 LMH, when the flow rate was increased from F1(600,300) to F2 (1000,400). The improvement in water flux resulted from the occurrence of turbulence at a higher flow rate or crossflow velocity that helped to mitigate the occurrence of DECP at the membrane surface by mixing the permeate water faster in the bulk DS [78]. However, the solute flux was higher for F1(600,300) flowrate and less for F2 (1000,400) flowrate. Significantly higher flux was achieved when using F3 (1500,600), and this is due the fact that the DS flow rate is lower and the osmotic gradient is higher, which corresponded to increased flux. With F3 (1500,600) flow rate, the water flux obtained was quite higher and uncontrollable. Also, the solute flux value was higher in F3 (1500,600) flow rate. Hence, it was decided to proceed with F2 (1000,400) flow rate for the remaining studies due to its higher water flux and lower solute flux.
